# Non-Coding RNAs in Asthma: Regulators of Eosinophil Biology and Airway Inflammation

**DOI:** 10.3390/diagnostics15141750

**Published:** 2025-07-10

**Authors:** Eglė Vasylė, Andrius Januškevičius, Kęstutis Malakauskas

**Affiliations:** 1 Laboratory of Pulmonology, Department of Pulmonology, Lithuanian University of Health Sciences, 44307 Kaunas, Lithuania; 2 Department of Pulmonology, Lithuanian University of Health Sciences, 44307 Kaunas, Lithuania

**Keywords:** asthma, eosinophil, airway inflammation, ncRNA, lncRNA, circRNA, miRNA, piRNA

## Abstract

Asthma is a complex and heterogeneous disease characterized by chronic airway inflammation, bronchial hyperresponsiveness, and reversible airflow obstruction. Despite extensive research, its underlying molecular mechanisms remain incompletely understood. Among the key immune cells involved, eosinophils play a central role in asthma pathophysiology through their contributions to Type 2 inflammation, tissue remodeling, and immune regulation. Recent studies have shown that non-coding RNAs (ncRNAs) play a crucial role in regulating eosinophil biology and contribute to the molecular mechanisms underlying asthma progression. This review consolidates the current understanding of ncRNAs in the development of eosinophils, their involvement in asthma pathogenesis, and the mechanisms underlying this process.

## 1. Introduction

Asthma is a common chronic inflammatory disease of the airways that affects a substantial proportion of the global population in different countries. The inflammatory response in asthma involves a wide range of specialized immune cells, including eosinophils, mast cells, neutrophils, T lymphocytes, and macrophages, as well as structural cells such as airway epithelial cells, fibroblasts, and bronchial smooth muscle cells. Their interplay results in excessive mucus secretion, smooth muscle contraction, airway wall remodeling, and ultimately, airway narrowing [1].

Among these immune cells, eosinophils have been identified as key effectors in driving airway inflammation and tissue damage, particularly in eosinophilic asthma phenotypes. Eosinophils are terminally differentiated granulocytes that play a critical role in asthma pathogenesis. These cells migrate from the bone marrow to the airways via the bloodstream, where they release a wide array of molecules that influence surrounding cells and regulate their own activity [2]. Accumulating evidence suggests that ncRNAs are also involved in regulating gene expression within eosinophils, thereby modulating their differentiation, survival, and effector functions. Beyond intracellular regulation, eosinophils secrete ncRNAs into the extracellular environment, primarily via exosomes, where they may influence neighboring structural or immune cells [3,4].

Importantly, ncRNAs are remarkably stable in serum and resistant to RNase degradation. This stability is partly due to their packaging into exosomes—lipid bilayer-enclosed vesicles that originate from endosomes. Exosomal membranes are enriched with phosphatidylserines, cholesterol, sphingolipids, and detergent-resistant lipid rafts, which protect their cargo from proteases and RNases during extracellular transport [3,5]. These features make ncRNAs attractive candidates for monitoring disease-associated cellular changes and as non-invasive biomarkers in asthma.

However, studies examining the role of ncRNAs, especially in key inflammatory cells such as eosinophils, remain limited. This area of research is underexplored, despite its potential to deepen our understanding of asthma pathogenesis and guide personalized treatment approaches. This review provides a comprehensive overview of the regulatory functions of ncRNAs in eosinophil differentiation, activation, and effector mechanisms, as well as the emerging roles of exosome-associated ncRNAs in asthma pathogenesis. In addition to extensively covering microRNAs (miRNAs) and long non-coding RNAs (lncRNAs), this review also integrates the latest findings on Piwi-interacting RNAs (piRNAs) and incorporates data from integrative omics approaches that reveal novel interactions between miRNAs and metabolic intermediates. By highlighting key mechanistic insights from recent experimental studies, this article offers perspectives on the complex molecular networks underlying asthma and discusses the diagnostic and therapeutic potential of ncRNAs. Finally, current challenges and future prospects for ncRNA-based therapies in asthma are critically evaluated, underscoring the potential of these molecules as next-generation biomarkers and therapeutic targets.

## 2. Non-Coding RNA

NcRNAs regulate target genes via multiple mechanisms and engage in mutual interactions, forming an intricate and adaptable RNA-based regulatory network. Initial genomic studies, primarily based on bacterial and yeast models, suggested that most functional DNA encodes proteins. However, data from the ENCODE project revealed that approximately 76% of the human genome is transcribed into RNA, while only about 1.2% of this transcribed RNA encodes proteins, with the remaining majority corresponding to ncRNAs [6,7]. Following their discovery, the biological importance of ncRNAs became increasingly evident, and they were recognized as key elements in gene regulation and genome structure.

**Figure 1:**
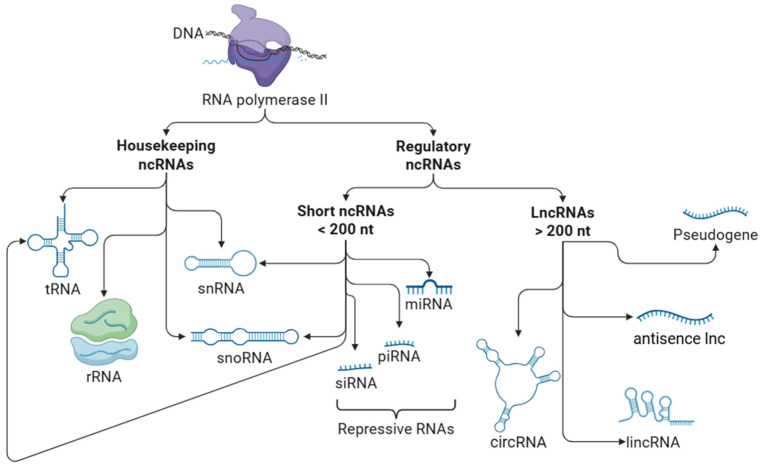
Types of non-coding RNAs. Non-coding RNAs (ncRNAs) are broadly categorized into two functional groups: housekeeping and regulatory ncRNAs. Housekeeping ncRNAs are constitutively expressed and are essential for fundamental cellular processes such as protein synthesis and RNA modification. This group includes ribosomal RNAs (rRNAs), transfer RNAs (tRNAs), small nuclear RNAs (snRNAs), and small nucleolar RNAs (snoRNAs). Although functionally distinct, tRNA, snRNAs, and snoRNAs are considered short ncRNAs based on their size (<200 nucleotides). Regulatory ncRNAs are involved in the modulation of gene expression at both transcriptional and post-transcriptional levels. They are further classified by length into short ncRNAs (<200 nucleotides), which include microRNAs (miRNAs), small interfering RNAs (siRNAs), and Piwi-interacting RNAs (piRNAs). These small ncRNAs often function as repressive molecules, silencing gene expression by promoting mRNA degradation or inhibiting translation. Long non-coding RNAs (lncRNAs) (>200 nucleotides) include long intergenic non-coding RNAs (lincRNAs), circular RNAs (circRNAs), antisense lncRNAs, and transcribed pseudogenes, the latter of which may act as functional RNAs with regulatory roles. Note: This figure presents a simplified classification of ncRNAs for illustrative purposes and does not include all subclasses.

NcRNAs are a group of RNAs without protein-coding functions. Based on their functional characteristics, ncRNAs are broadly categorized into two main types: housekeeping ncRNAs, which are involved in essential cellular processes, and regulatory ncRNAs, which modulate gene expression. Regulatory ncRNAs can be further classified by length into short ncRNAs (<200 nucleotides) and lncRNAs (>200 nucleotides) (Figure 1).

The altered expression of a single ncRNA may modulate the activity of additional ncRNAs, ultimately affecting multiple cellular functions, including transcriptional regulation, RNA processing, transport, and protein synthesis [8].

### 2.1. Small Non-Coding RNAs

This group includes miRNAs, piRNAs, small interfering RNAs (siRNAs), small nuclear RNAs (snRNAs), small nucleolar RNAs (snoRNAs), and transfer RNAs (tRNAs).

#### 2.1.1. Transfer RNAs

tRNAs are small ncRNA molecules that play an essential role in protein synthesis by delivering specific amino acids to the ribosome. Each tRNA contains an anticodon sequence that pairs with the corresponding codon on the messenger RNA (mRNA), ensuring the correct incorporation of amino acids into the growing polypeptide chain during translation [9].

#### 2.1.2. Small Nuclear RNAs

snRNAs are a class of short, ncRNA molecules typically ranging from 80 to 350 nucleotides in length. They are predominantly localized in the nucleoplasm of eukaryotic cells, particularly within splicing speckles and Cajal bodies [10]. snRNAs are often referred to as U-RNAs due to their uridine-rich sequences and are transcribed by either RNA polymerase II or III, depending on the subtype. Functionally, snRNAs are best known for their essential role in pre-mRNA intron splicing, as they constitute the core RNA components of spliceosomes—large ribonucleoprotein complexes, together with other proteins, responsible for intron removal and exon ligation. In addition to their splicing function, certain snRNAs are also involved in regulating transcriptional activity, including the control of transcription factors and RNA polymerase II [10,11].

#### 2.1.3. Small Nucleolar RNAs

snoRNAs are a class of small ncRNAs, typically 70–200 nucleotides in length. They are localized in the nucleolus and Cajal bodies of eukaryotic cells. Most snoRNAs are encoded within the introns of protein-coding genes and are processed during splicing [12]. Some snoRNAs can be further processed into smaller fragments known as snoRNA-derived RNAs, a subset of which resemble miRNAs in both size and function [13]. Traditionally, snoRNAs are known to guide site-specific modifications of ribosomal RNAs (rRNAs), such as 2′-O-methylation and pseudouridylation—processes essential for ribosome biogenesis [11,14]. However, recent research has revealed a broader functional repertoire. snoRNAs have also been implicated in RNA acetylation, the modification of mRNAs and tRNAs, the regulation of RNA editing and alternative splicing, as well as the modulation of protein stability through direct RNA–protein interactions [15,16,17,18].

#### 2.1.4. PIWI-Interacting RNAs

Although numerous classes of small ncRNAs have been identified, those with regulatory functions are generally categorized into three major groups based on their biological functions, associated effector proteins, origins, and structural features: piRNAs, siRNAs, and miRNAs. These three eukaryotic small ncRNAs typically range in size from 20 to 31 nucleotides in length. All three classes function through interactions with members of the Argonaute protein family, which is divided into two subfamilies: AGO and PIWI proteins. miRNAs and siRNAs are associated with AGO proteins to form RNA-induced silencing complexes (RISCs) and are processed from double-stranded RNA precursors in a Dicer-dependent manner. In contrast, piRNAs are generated from long single-stranded precursor transcripts derived from intergenic genomic regions known as piRNA clusters and bind to PIWI proteins, primarily expressed in germline cells, forming piRNA-induced silencing complexes (piRISCs). These complexes mediate post-transcriptional gene silencing and transposon repression through Dicer-independent mechanisms [19,20].

piRNAs are typically 24–31 nucleotides in length that are characterized by a 2′-O-methyl modification at their 3′ end [21,22]. These clusters are rich in transposable element sequences, and piRNAs primarily function to silence transposons, thereby preserving genome integrity, especially during gametogenesis and reproduction. Because uncontrolled transposition can threaten genome stability, the piRNA pathway acts as a defense system, conceptually similar to an innate immune mechanism, enabling the cell to distinguish between “self” and “non-self” genetic elements [23]. piRNAs are the most diverse class of small RNAs and represent the largest population of ncRNAs in animal cells [22,24]. In addition to transposon silencing, emerging studies suggest that some piRNAs also regulate protein-coding genes and may participate in epigenetic inheritance, transmitting information about transposon activity to subsequent generations [25,26,27].

#### 2.1.5. Small Interfering RNAs

siRNAs are double-stranded RNA molecules, approximately 21–23 nucleotides in length, that suppress gene expression through the RNA interference pathway [28,29]. Following their biogenesis, siRNAs are incorporated into the RISC, where the duplex is unwound and one strand is retained as a guide to direct sequence-specific binding to complementary mRNA. This interaction results in the cleavage and subsequent degradation of the target mRNA, thereby preventing its translation into protein. Functionally, siRNAs share similarities with miRNAs, as both can inhibit gene expression by promoting mRNA degradation or by repressing translation [28].

#### 2.1.6. MicroRNAs

miRNAs are small, single-stranded ncRNAs, 20–25 nucleotides in length, that regulate gene expression at the post-transcriptional level [30]. Most miRNAs are transcribed by RNA polymerase II or III into primary transcripts (pri-miRNAs), which are processed into precursor miRNAs (pre-miRNAs) and ultimately into mature miRNAs [31]. Nearly half of all identified miRNAs are intragenic, primarily derived from introns and relatively few exons of protein-coding genes, while the rest are intergenic and transcribed independently under their own promoters [32,33]. In some cases, multiple miRNAs are transcribed as clusters, often sharing similar seed sequences and forming functional families [34].

Following maturation, miRNAs are incorporated into the RISC, where they guide the complex to target mRNAs through complementary base-pairing. Most commonly, miRNAs bind to the 3′ untranslated regions (3′ UTRs) of their targets, leading to translational repression or mRNA degradation [31]. However, interactions with other regions, including the 5 ′ UTR, coding sequences, and even gene promoters, have also been reported [35]. Interestingly, under specific cellular conditions, such as cell cycle arrest, nutrient deprivation, or quiescence, certain miRNAs have been shown to activate gene expression rather than repress it. This activation is mediated by AGO2 and FXR1 (instead of GW182), which associate with AU-rich elements in the 3′ UTR or, in some cases, with the 5′ UTR of mRNAs encoding ribosomal proteins during amino acid starvation [36,37,38,39]. Recent studies further suggest that miRNAs are actively transported between subcellular compartments, where they may regulate not only translation but also transcriptional activity [40]. A single miRNA can regulate multiple genes, while a single gene may be targeted by several different miRNAs, thus influencing key cellular regulatory networks [41]. Consequently, aberrant miRNA expression has been implicated in the initiation and progression of various human diseases, including asthma.

### 2.2. Long Non-Coding RNAs

LncRNA transcripts are longer than 200 nucleotides and lack significant protein-coding ability. This group encompasses long intergenic ncRNAs (lincRNAs), antisense lncRNAs, circular RNAs, and pseudogenes. Some lncRNAs may contain short open reading frames (sORFs) [42]. sORFs are nucleotide sequences between a start and stop codon that are typically less than 300 nucleotides in length. These sORFs are usually considered non-functional, but recent studies suggest that, in certain cases, they may encode bioactive micropeptides, indicating that some transcripts previously classified as non-coding might have coding potential [43]. Most annotated lncRNAs are transcribed by RNA polymerase II; therefore, they share structural similarities with mRNAs, such as 5′ cap structures and poly(A) tails. LncRNAs are present both in the nucleus and cytoplasm and can interact with DNA, RNA, or proteins, thereby modulating transcription, post-transcriptional regulation, and epigenetic processes [42]. Although structurally similar to mRNAs, lncRNAs differ by being shorter, containing fewer but longer exons, and showing higher tissue specificity. Importantly, lncRNAs may act as competitive endogenous RNAs, modulating gene expression by binding to miRNAs. Regulation between lncRNAs and miRNAs (either through direct interaction or via other molecular mediators) adds further complexity to the post-transcriptional regulatory network [44].

#### 2.2.1. Long Intergenic Non-Coding RNAs

LincRNAs are transcribed from genomic regions that do not overlap with protein-coding genes. They represent more than half of all lncRNA transcripts in humans. LincRNAs exert diverse regulatory functions, including chromatin remodeling, genome architecture modulation, transcriptional regulation (including enhancer-associated activity—this arises from transcription at enhancer-like regions that influence nearby gene expression), and RNA stabilization [45,46,47]. Although generally considered non-coding, a few annotated lincRNAs contain sORFs that may encode functional small peptides [48].

#### 2.2.2. Antisense Long Non-Coding RNAs

Antisense lncRNAs are transcribed from the strand opposite to protein-coding or non-coding genes and are defined based on their genomic orientation relative to neighboring genes [49]. They regulate gene expression either in cis, by perfect base-pairing with the promoter of their overlapping gene, or in trans, by imperfect sequence binding to other gene targets [50]. Nuclear antisense lncRNAs often affect transcription via histone or DNA modifications, recruiting specific factors to the DNA at the transcriptional level, while cytoplasmic antisense lncRNAs can regulate mRNA stability, translation, or act as competing endogenous RNAs by sponging miRNAs. Furthermore, cytoplasmic antisense lncRNAs can bind to proteins, altering their half-lives [51].

#### 2.2.3. Circular RNAs

CircRNAs are covalently closed, single-stranded RNA molecules that lack 5′ caps and 3′ poly(A) tails, making them highly resistant to exonucleases and remarkably stable within cells [52]. Their half-life often exceeds 48 h, significantly longer than that of linear mRNAs, which typically have a half-life of ~10 h [53]. Their expression is often cell-type and tissue-specific. While exonic circRNAs are typically cytoplasmic, intronic circRNAs and intron retained circRNAs are often nuclear and regulate transcription or splicing of their parental genes [54]. Functionally, circRNAs regulate gene expression by acting as miRNA sponges, modulators of transcription, scaffolds for protein complexes, decoys for RNA-binding proteins, or play critical roles in innate immune pathways [55,56,57,58,59]. Some circRNAs have even been shown to be translatable under specific conditions via internal ribosome entry sites [60,61].

#### 2.2.4. Pseudogenes

Pseudogenes are DNA sequences that closely resemble their parental genes but have lost their ability to encode functional proteins due to disabling mutations and were long regarded as non-functional “junk DNA” [62]. Depending on their origin, they are classified as processed, unprocessed, or unitary pseudogenes [63,64]. Many pseudogenes function as lncRNAs, regulating gene expression through diverse mechanisms, including miRNA sponging and RNA-binding protein sequestration [65]. Due to their high sequence similarity with parental genes, pseudogene-derived lncRNAs can compete with endogenous transcripts, thereby modulating gene expression networks. Furthermore, some pseudogenes interact with epigenetic regulators, contributing to chromatin remodeling and transcriptional silencing [66,67].

## 3. Eosinophil Biology

Eosinophils arise from hematopoietic stem cells in the bone marrow and represent one branch of the white blood cell lineage. During eosinopoiesis, cells sequentially transition from common myeloid progenitors through eosinophil-committed progenitors, myeloblasts, promyelocytes, and myelocytes, ultimately yielding mature eosinophils [68]. This maturation is orchestrated by transcription factors such as GATA-1, GATA-2, PU.1, C/EBP, IRF8, FOG-1, and XBP1, and is driven by the cytokines granulocyte–macrophage colony-stimulating factor (GM-CSF), interleukin (IL)-3, and IL-5 [69]. Granule biogenesis begins at the promyelocyte stage, where primary (early secondary) granules form [70]. Subsequently, in the myelocyte stage, they generate the distinctive crystalloid or secondary granules characteristic of mature eosinophils [71]. These granules facilitate the storage of four preformed toxic cationic proteins: major basic protein-1 (MBP-1), eosinophil peroxidase (EPX), eosinophil-derived neurotoxin (EDN), and eosinophil cationic protein (ECP), along with a broad range of chemokines, cytokines, and growth factors [69,72,73]. Once released into the bloodstream, eosinophils are recruited to tissues such as the lungs, thymus, mammary glands, uterus, and gastrointestinal tract in response to eotaxins and other chemokines [74,75,76]. Increasing evidence suggests that they may enter tissues in a partially mature state or even differentiate locally through in situ hematopoiesis [77]. Although their circulatory half-life is only 8–18 h, eosinophils can extend their survival in tissues via interactions with resident cells and supportive cytokines [68,78].

Eosinophils contribute to host defense against bacteria, helminths, parasites, and viruses, yet they also play a central role in the inflammation and tissue remodeling seen in allergic and non-allergic diseases, particularly those marked by eosinophil-rich infiltrates or extracellular deposition of their granule proteins, which may result in local inflammation, tissue damage, remodeling [69,79,80,81]. Despite decades of study, however, the full spectrum of eosinophil functions in health and disease remains an active area of research.

### 3.1. Eosinophils’ Development Control by Non-Coding RNA

miRNAs fine-tune hematopoietic processes that govern eosinophil lineage commitment. miR-223 regulates eosinophil development by targeting the insulin-like growth factor 1 receptor (IGF1R), a key mediator of cell proliferation and survival. During eosinophilopoiesis, miR-223 expression is upregulated, directly suppressing IGF1R, which limits the proliferation of progenitor cells and promotes proper differentiation. In miR-223^−^/^−^ (knockout) mouse models, elevated IGF1R levels lead to increased eosinophil progenitor proliferation and delayed maturation, as indicated by postponed CCR3 surface expression [82]. Notably, elevated miR-223 levels have been observed in asthma and other eosinophilic disorders [83,84] possibly reflecting a compensatory mechanism that counteracts excessive eosinophil expansion driven by enhanced IGF1R signaling.

miR-21 is also upregulated during eosinophil progenitor development and plays a critical role in promoting cell proliferation and survival. In miR-21^−^/^−^ eosinophil progenitor cultures, proliferation is reduced and apoptosis is increased, potentially due to derepression of anti-proliferative and pro-apoptotic targets such as PDCD4, PTEN, and Psrc1, the latter being a predicted direct target of miR-21 [83,85]. Consistently, in vivo miR-21^−^/^−^ mice exhibit reduced blood eosinophil counts and diminished colony-forming capacity in bone marrow [85].

In addition to miR-21, the complementary passenger strand miR-21* was also found to be upregulated in eosinophils upon GM-CSF stimulation, mimicking the elevated cytokine environment of inflamed airways. This upregulation contributed to prolonged eosinophil survival by enhancing intracellular ERK phosphorylation, suggesting that miR-21* protects eosinophils from apoptosis via ERK pathway activation. Functional assays demonstrated that transfection with miR-21* mimics significantly reduced spontaneous apoptosis in vitro, while inhibition of miR-21* suppressed GM-CSF-induced ERK activation and decreased eosinophil viability. These findings support a key immunomodulatory role for miR-21* in promoting eosinophil persistence under inflammatory conditions, such as those observed in allergic airway inflammation [86].

Furthermore, EGO (eosinophil granule ontogeny)—an lncRNA transcribed from a conserved intronic region of the *inositol triphosphate receptor type 1* (*ITPR1*) gene—is rapidly upregulated upon IL-5 stimulation in CD34^+^ hematopoietic progenitor cells. EGO lacks significant ORFs, and the sORFs present poor amino acid conservation, further supporting its classification as an ncRNA. EGO is not associated with ribosomes and localizes to non-polysomal fractions, indicating that it is not translated into protein. Functional experiments revealed that EGO silencing in CD34^+^ hematopoietic progenitor cells led to a significant decrease in the expression of eosinophil granule protein transcripts, including MBP and EDN, without affecting the expression of GATA-1, a key transcription factor in eosinophil development, indicating a GATA-1-independent regulatory pathway. Although the exact mechanism remains unclear, it has been proposed that EGO may act as siRNA or alternatively function through RNA–protein complexes that either influence transcriptional activity or stabilize specific mRNAs. The inducible expression of EGO during IL-5-driven differentiation and its bone marrow specificity support its functional importance in eosinophil granule maturation and eosinophilopoiesis [87].

### 3.2. Functional Modulation of Eosinophil Activity

Among the eosinophil-enriched lncRNAs identified in transcriptomic databases, ITGB2-AS1 (ITGB2 Antisense RNA 1) has emerged as a particularly relevant molecule with regulatory potential in eosinophilic disorders. In vitro studies using the human promyelocytic leukemia cell line HL-60 clone 15 cells (HL-60c15) demonstrated that stable knockdown of ITGB2-AS1 impairs eosinophil differentiation. Specifically, silencing of this lncRNA led to a reduction in cytoplasmic granule formation and decreased expression of eosinophil-specific proteins, including EPX and MBP-1, both of which are essential for eosinophil cytotoxic function. Moreover, the cell surface expression of CCR3 (a chemokine receptor crucial for eosinophil recruitment and a marker of terminal maturation) was significantly downregulated in ITGB2-AS1-deficient cells, suggesting a developmental arrest at the myelocyte stage. Additionally, CCR1 (another chemokine receptor implicated in eosinophil activation and migration) was also markedly reduced, indicating a broader impairment in the functional maturation of eosinophils, which reinforces the functional impact of ITGB2-AS1 knockdown [88]. Furthermore, ITGB2-AS1-deficient cells showed downregulation of ITGB2 (also known as CD18)—a β-subunit of leukocyte integrins essential for eosinophil interactions with lymphocytes, adhesion to airway epithelial cells, and chemokine-mediated tissue infiltration [88,89,90,91]. This was associated with reduced reactive oxygen species (ROS) production and impaired degranulation capacity [88]. These findings suggest that the lncRNA ITGB2-AS1 functions not only as a regulator of eosinophil differentiation but also as a modulator of mature eosinophil effector activity.

Furthermore, in mature eosinophils, Morrbid (MyelOid Rna Regulator of Bim-Induced Death) acts as a chromatin-associated lncRNA that controls apoptosis by epigenetically repressing the pro-apoptotic gene *Bcl2l11* (*Bim*). Knockdown of Morrbid leads to increased *Bcl2l11* expression, elevated apoptosis, and reduced eosinophil viability [92]. Notably, in hypereosinophilic syndrome, where IL-5 levels are elevated [93], Morrbid expression in eosinophils correlates positively with plasma IL-5 [92], suggesting a role in cytokine-driven eosinophil persistence in inflammatory diseases.

Gene silencing by siRNA has emerged as a powerful tool in immunological research. In a murine model of chronic allergic asthma, the intranasal administration of a commercially designed suppressor of cytokine signaling 3 (SOCS3) siRNA resulted in the attenuation of airway inflammation and remodeling by reducing eosinophilia, mucus secretion, and airway hyperresponsiveness [94]. Extending this approach to human eosinophils, SOCS3 silencing revealed its pivotal role in modulating eosinophil functional properties. Treatment with SOCS3 siRNA pool (s17190, s17191, s17189) suppressed Th2-associated transcription factors (GATA-3, FoxP3) and IL-10 expression, thereby diminishing CCR3 expression and impairing eosinophil migration toward IL-5, eotaxin, and fMLP gradients. Adhesion was also attenuated, likely via downregulation of VLA-4 and LFA-1 integrins. Interestingly, SOCS3 knockdown increased cytolytic degranulation upon Th2 cytokine stimulation, potentially leading to premature eosinophil death and the release of toxic granules into the circulation. These findings suggest that while SOCS3 supports Th2 polarization and effector function, its overexpression in asthma may exacerbate eosinophil-driven inflammation, indicating that fine-tuning rather than complete suppression of SOCS3 (to avoid massive degranulation) may offer therapeutic benefits [95].

### 3.3. Eosinophil-Derived Exosomes and Their Functional Roles in Asthma

Eosinophils contribute to asthma pathophysiology by releasing cationic proteins (MBPs, EPOs, ECPs, and EDNs), chemokines (RANTES and eotaxin-1), pro-inflammatory cytokines (IL-2, IL-4, IL-5, IL-10, IL-12, IL-13, IL-16, IL-18, and transforming growth factor (TGF)-α/β), and lipid mediators (LTC4, platelet-activating factor, thromboxane B2, and prostaglandins) [96,97]. These molecules are packaged and stored in eosinophil intracellular granules and can be rapidly released into the extracellular environment to act on other cells or on eosinophils themselves [98]. In addition to classical degranulation, eosinophils can release extracellular vesicles such as exosomes, which originate from the endosomal compartment and carry regulatory molecules, including ncRNAs.

Eosinophils release exosomes approximately 162 nm in size (as measured by NanoSight), with a cup-shaped morphology, containing CD63, CD9, and ALIX proteins, which confirms their endosomal origin. Exosomes are abundantly found in body fluids, including blood, tears, urine, saliva, and breast milk. According to studies, the presence of integrin CD47 on the exosomal surface protects these vesicles from phagocytosis by monocytes and macrophages [99]. These vesicles generally carry nucleic acids (DNA and RNA), proteins, and lipid mediators. In addition, eosinophil-derived exosomes contain cytotoxic granule proteins such as MBP, EPO, and ECP, which can contribute to tissue damage and exacerbate asthma symptoms. Although eosinophil-derived exosomes from healthy and asthmatic individuals share a broadly similar composition, eosinophils from asthmatic patients secrete a significantly higher number of exosomes [2]. This increased exosomal load, together with potential alterations in molecular cargo, may amplify their biological effects in the airways. Mediators contained in eosinophil-derived exosomes enhance the production of ROS and nitric oxide, promote eosinophil migration, increase adhesion and expression of adhesion molecules, and ultimately contribute to airway epithelial injury and asthma exacerbation. Studies indicate that exosomal ncRNAs play important roles in the progression of pulmonary diseases, including asthma, chronic obstructive pulmonary disease, and tuberculosis [100].

Cañas et al. demonstrated that eosinophil-derived exosomes from asthmatic patients influence structural lung cells, including small airway epithelial cells and bronchial smooth muscle cells [101]. These exosomes increase apoptosis and impair wound repair in airway epithelial cells. They also upregulate the expression of pro-inflammatory genes, such as *TNF*, *CCL26* (*eotaxin-3*), and *POSTN* (*periostin*), which contribute to sustained inflammation and airway remodeling. Mechanistically, the exosomes reduce the phosphorylation of STAT3 (a key transcription factor involved in inflammation and epithelial repair) and AKT (factor for the regulation and blockade of apoptosis and promotion of cell survival) in airway epithelial cells, leading to increased apoptosis and decreased epithelial cell motility and wound repair—hallmarks of impaired epithelial regeneration [101,102,103]. Meanwhile, eosinophil-derived exosomes promote the proliferation of bronchial smooth muscle cells via the MAPK/ERK signaling pathway. Stimulation with exosomes from asthmatic eosinophils also enhances *CCR3* and *VEGFA* gene expression: *CCR3* activation initiates proliferation through the MAPK cascade, while *VEGFA* is associated with bronchial wall remodeling, a characteristic of asthma [101,104]. These effects are absent when using exosomes from healthy donors, suggesting that disease-specific alterations in exosome abundance or cargo contribute to asthma pathogenesis.

The presence of miRNAs in exosomes from bodily secretions has been confirmed in studies identifying 24 miRNAs in bronchoalveolar lavage fluid. Levels of these miRNAs were closely associated with pulmonary function, particularly forced expiratory volume in one second (FEV1). Furthermore, several miRNAs were shown to influence cytokine levels, including IL-6, IL-13, IL-10, and IL-8. The expression of certain miRNAs, such as those from the let-7 family and the miR-200 family, miR-203, miR-130, and miR-125b were found to be significantly downregulated in exosomes from patients with mild asthma compared to healthy controls [100].

Given their stability and accessibility in body fluids, eosinophil-derived exosomal ncRNAs are increasingly recognized as promising non-invasive biomarkers for asthma diagnosis and monitoring. Moreover, there is growing interest in targeting the exosomal RNA cargo for therapeutic purposes. Modulating the ncRNA content of exosomes may represent a novel strategy for asthma treatment. However, current data on the specific roles of exosomal lncRNAs in asthma pathogenesis remain limited.

## 4. Non-Coding RNAs in the Regulation of Airway Inflammation

Asthma is a heterogeneous disease characterized by variable airway obstruction and increased bronchial hyperresponsiveness, prompting the search for novel therapeutic targets. Various studies have demonstrated the functional involvement of ncRNAs in asthma pathogenesis (see Table 1). For example, altered expression of miR-371a-5p has been linked to bronchiolitis, asthma, and chronic obstructive pulmonary disease [105]. Increased expression of miR-16 has been observed in the mice airways exposed to house dust mite allergens, while inhibition of miR-145 and miR-106a has been found to reduce allergen-induced airway inflammation. Additionally, miR-570 has been shown to regulate the expression of the RNA-binding protein HuR, a molecule involved in sustaining chronic airway inflammation in asthma [106]. Elevated levels of miR-485-3p and miR-221 have also been reported in the blood of children with asthma compared to healthy controls [107]. Furthermore, miR-1246, miR-320a, miR-21-5p, and miR-629-5p has negatively correlated with forced vital capacity (FVC) in asthma patients, with miR-629-5p alone negatively correlating with forced expiratory volume in one second (FEV_1_) [108].

In addition to miRNAs, recent studies have emphasized the significance of circRNAs in modulating immune responses in asthma. For example, circ_0002594 competitively suppresses multiple miRNAs (miR-16-5p, miR-503-5p, miR-514a-3p, miR-587, and let-7e-5p), which may prove valuable for the diagnosis and treatment of Th2-type asthma [109]. Moreover, the upregulation of circ_0000629 and circ_0000455 enables them to interact with miR-15a and miR-29b, miRNAs known to negatively regulate allergic inflammation [110,111]. miR-29b facilitates Th2 cytokine production and eosinophilic inflammation by targeting inducible costimulatory factors [112], while miR-15a is decreased in Th2-mediated airway inflammation, which leads to increased endothelial growth factor (VEGF) expression and promotes airway remodeling in asthma [113]. Conversely, regulatory networks involving downregulated circRNAs such as circ_0000723 and circ_0001454, together with their respective miR-214 and miR-146b targets, have been linked to increased hypersensitivity, highlighting the intricate roles of circRNAs and miRNAs in asthma pathogenesis [114].

**Table 1:** Comprehensive summary of non-coding RNAs in airway inflammation.

ncRNA Name (type)	Target Cells/ Affected Cells	Main Target(s)/Gene(s)	Role/Function	Upregulated/Downregulated	Reference
miR-223 (miRNA)	Eosinophil progenitor cells	IGF1R	Regulates eosinophil development; limits progenitor proliferation and promotes differentiation	Upregulated during eosinophilopoiesis and in asthma/eosinophilic disorders	[82,83,84]
miR-21 (miRNA)	Eosinophil progenitors	PDCD4, PTEN, Psrc1 (predicted)	Promotes cell proliferation and survival, highest expression in eosinophilic asthma. Antagomir therapy inhibits Th2 activation	Upregulated during eosinophil development and in allergic airway inflammation	[83,85]
	Plasma	Not specified	Specific marker for eosinophilic asthma	Increase stepwise from noneosinophilic to eosinophilic asthma	[115]
miR-21 *	Mature eosinophils (GM-CSF stimulated)	ERK signaling pathway	Prolongs eosinophil survival and protects from apoptosis	Upregulated upon GM-CSF stimulation	[86]
miR-203, miR-130, miR-125b (miRNAs)	Not specified	Not specified	Not specified	Downregulated in mild asthma	[100]
miR-371a-5p (miRNA)	Not specified	Not specified	Linked to bronchiolitis, asthma, COPD	Differentially increased in bronchiolitis, asthma, COPD	[105]
miR-16 (miRNA)	Mouse airway (HDM model)	Not specified	Not specified	Upregulated in allergen-exposed airways	[106]
miR-570 (miRNA)	Airway epithelial cells	HuR (ELAVL1)	Sustain chronic inflammation	Upregulated upon TNFα stimulation	[106]
miR-221 (miRNA)	Not specified	Not specified	Not specified	Upregulated in the blood of children with asthma	[107]
	Airway smooth muscle cells	Not specified	Regulates airway smooth muscle cell proliferation and IL-6 production	Upregulated in severe asthma	[116]
miR-485-3p (miRNA)	Not specified	Not specified	Not specified	Upregulated in the blood of children with asthma	[107]
miR-1246, miR-320a, miR-21-5p (miRNAs)	Not specified	Not specified	Negatively correlated with FVC	Upregulated in eosinophils in asthma	[108]
miR-629-5p (miRNA)	Not specified	Not specified	Negatively correlated with FEV_1_	Upregulated in eosinophils in asthma	[108]
miR-29b (miRNA)	Ovalbumin-induced murine	ICOS	Facilitates eosinophilic inflammation	Downregulated in ovalbumin-induced asthmatic mice	[112]
miR-15a (miRNA)	Not specified	VEGF	Promotes an asthma-like phenotype	Downregulated in Th2 airway inflammation	[113]
let-7 family (miRNA)	Not specified	Not specified	Not specified	Downregulated in mild asthma	[100]
	Not specified	Not specified	Reflect asthma severity	Increase stepwise from mild to severe asthma	[115]
miR-98 (miRNA)	Not specified	Not specified	Reflect asthma severity	Increase stepwise from mild to severe asthma	[115]
miR-155 (miRNA)	Plasma	Not specified	Specific marker for eosinophilic asthma	Increase stepwise from noneosinophilic to eosinophilic asthma and from mild to severe asthma	[115,117]
	Bronchial epithelial cells	Not specified	Not specified	Increased in bronchial epithelial cells in asthma	[117]
miR-185-5p (miRNA)	Serum	Not specified	Reflect asthma severity	Increase stepwise from mild to severe asthma	[108]
miR-19a (miRNA)	CD4^+^ T cells; ILC2s	Not specified	Stimulates IL-13 secretion; promotes CD4^+^ T cell survival/proliferation; drives cytokine production in ILC2s	Upregulated in asthma	[118,119,120]
miR-126 (miRNA)	CD4^+^ T cells	Not specified	Indirectly upregulates GATA-3, enhancing Th2 cytokine (IL-5, IL-13) secretion	Upregulated in asthma	[121]
miR-24, miR-27 (miRNAs)	CD4^+^ T cells	Not specified	Inhibit IL-4 production in CD4^+^ T cells	Not specified	[122]
miR-371, miR-138, miR-544, miR-145, miR-214 (miRNAs)	CD4^+^ T cells	Runx3	Shifting Th1/Th2 balance toward Th2	Upregulated in asthma	[123]
miR-1 (miRNA)	Bronchial smooth muscle cells	Not specified	Loss of miR-1 is associated with smooth muscle cell hypertrophy	Downregulated in asthma	[124]
miR-26a (miRNA)	Airway smooth muscle cells	GSK-3β	Promotes hypertrophic signaling	Upregulated upon mechanical stretch	[125]
miR-200 family (miRNA)	Not specified	Not specified	Not specified	Downregulated in mild asthma	[100]
	Airway epithelial cells	ZEB1 via ERK/p38 pathway	Controls epithelial–mesenchymal transition	Downregulated	[126]
miR-146b-5p (miRNA)	Airway smooth muscle cells	Not specified	Not specified	Upregulated in airway smooth muscle cells in asthma upon stimulation	[127]
EGO (lncRNA)	CD34^+^ hematopoietic progenitors	MBP, EDN transcripts	Regulates eosinophil granule protein expression during differentiation	Upregulated upon IL-5 stimulation	[87]
ITGB2-AS1 (lncRNA)	Eosinophils (HL-60c15 cell model)	EPX, MBP-1, CCR3, CCR1, ITGB2 (CD18)	Regulator of eosinophil differentiation and mature eosinophil effector functions (degranulation, ROS production)	Not specified	[88,89,90,91]
Morrbid (lncRNA)	Mature eosinophils	Bcl2l11 (Bim)	Controls apoptosis by epigenetically repressing the pro-apoptotic gene	Upregulated in hypereosinophilic conditions	[92]
PVT1 (lncRNA)	Airway smooth muscle cells	c-MYC (transcription factor)	Reflect asthma severity and steroid resistance; regulates cellular proliferation and IL-6 release	Increase stepwise from mild to severe asthma and corticosteroid resistance	[128]
CASC7 (lncRNA)	Airway smooth muscle cells	Sponges miR-21 to increase PTEN expression	Enhances corticosteroid responsiveness by inhibiting the PI3K/AKT signaling pathway	Downregulated in airway smooth muscle cells from patients with severe asthma	[129]
GAS5 (lncRNA)	Airway smooth muscle cells, airway epithelium	Acts as a glucocorticoid receptor; sponges miR-10a	Regulates airway smooth muscle cell proliferation; reduction decreases airway hyperresponsiveness	Upregulated by pro-inflammatory mediators	[130,131]
MEG3 (lncRNA)	T cells (Treg/Th17)	Sponges miR-17, indirectly influencing FoxP3 and RORγt	Disrupts the Treg/Th17 balance, contributing to neutrophilic asthma	Not specified	[132]
LNC_000127 (lncRNA)	Blood	TCR/STAT/GATA3 signaling pathway	Modulator of Th2 inflammation; may enhance Th2 cytokine production	Upregulated in eosinophilic asthma compared to neutrophilic asthma and controls	[133]
fantom3_9230106C11 (lncRNA)	Th2 cells	GATA-1; miR-19 (predicted)	Th2 immune responses control	Downregulated in Th2 cells	[134]
MM9LINCRNAEXON12105+, AK089315 (lncRNA)	Th2 cells	genes involved in IL-4, IL-5, and IL-13 signaling, STAT5, STAT6, CCL17, CCL22	Th2-type inflammation regulation	Upregulated upon the induction of asthma	[135]
RP11-401.2 (lncRNA)	Th2 cells	Not specified	Not specified	Upregulated in asthma	[136]
ENST00000444682, ENST00000566098, and ENST00000583179 (lncRNAs)	CD4^+^ T cells	SMAD7, WNT2B, C/EBP, T-bet, NF-κB genes	Modulates Th2 differentiation and pro-inflammatory cytokine production	Upregulated in asthma	[137]
ENST00000579468 (lncRNAs)				Downregulated in asthma	
LNC_00882 (lncRNA)	Airway smooth muscle cells	Wnt/β-catenin signaling through sponging of miRNA-3619-5p	Promotes cell proliferation	Upregulated after platelet-derived growth factor stimulation	[138]
MALAT1 (lncRNA)	Airway smooth muscle cells	Sponges miRNA-150, which targets eIF4E (AKT pathway)	Promotes cell proliferation and migration	Upregulated after plate-let-derived growth factor exposure and in neonatal rat asthmatic models	[139,140]
NORAD (lncRNA)	Bronchial epithelial cells	Sponges miR-410-3p to regulate RCC2 and the Wnt/β-catenin pathway.	Modulates epithelial–mesenchymal transition, airway remodeling, and inflammation	Upregulated in TGF-β1-induced bronchial epithelial cells and in ovalbumin-challenged asthmatic mice	[141]
AK085865 (lncRNA)	Macrophages	Drives M2 polarization	Drives M2 macrophage polarization, which enhances ILC2 differentiation and amplifies type 2 inflammation	Upregulation in a murine asthma model	[142]
BAZ2B (lncRNA)	Macrophages	BAZ2B pre-mRNA	Promotes M2 macrophage activation and inflammation	Upregulated in asthmatic children	[143]
PTPRE-AS1 (lncRNA)	Macrophages	WDR5	Negative regulator of M2 polarization and M2-mediated inflammation	Downregulated in asthma	[144]
circ_0002594 (circRNA)	CD4+ T cells	miR-16-5p, miR-503-5p, miR-514a-3p, miR-587, let-7e-5p (predicted)	Positively correlated with FeNO	Upregulated in Th2 allergic asthma	[109]
piR-43770 (piRNA)	Not specified	Not specified	Associated with total serum IgE; involved T cell proliferation	Not specified	[145]
piR-58469 (piRNA)	Not specified	Not specified	Associated with total serum IgE; may indicate mitochondrial dysfunction; involved T cell proliferation	Not specified	[145]
piR-43768 (piRNA)	Not specified	Not specified	Associated with total serum IgE; involved T cell proliferation	Not specified	[145]
piR-33487 (piRNA)	Not specified	Not specified	Associated with eosinophils count; involved in thymus development	Not specified	[145]
piR-36063 (piRNA)	Not specified	Not specified	Associated with eosinophils count; involved in thymus development	Not specified	[145]
piR-32571 (piRNA)	Not specified	Not specified	Associated with eosinophils count; involved in thymus development	Not specified	[145]
piR-37213 (piRNA)	Not specified	Not specified	Associated with total serum IgE and eosinophils count; involved in thymus development and T cell proliferation	Not specified	[145]
piR-31038 (piRNA)	Not specified	Not specified	Associated with total serum IgE and eosinophils count; involved in thymus development and T cell proliferation	Not specified	[145]
piR-33520 (piRNA)	Not specified	Not specified	Associated with total serum IgE and eosinophils count; involved in thymus development and T cell proliferation	Not specified	[145]
piR-34021 (piRNA)	Not specified	Not specified	Associated with total serum IgE and eosinophils count; involved in thymus development and T cell proliferation	Not specified	[145]
piR-33064 (piRNA)	Not specified	Not specified	Affects epithelial tight junction integrity	Not specified	[145,146]
piR-57460 (piRNA)	Not specified	Not specified	May regulate mitochondrial apoptosis	Not specified	[145]
piR-36707 (piRNA)	Not specified	Not specified	May indicate mitochondrial dysfunction	Not specified	[145]
piR-35549 (piRNA)	Not specified	Not specified	May indicate mitochondrial dysfunction	Not specified	[145]

Note: * Indicates passenger miRNA strand.

### 4.1. Non-Coding RNAs Expression Patterns Across Asthma Severity

Assessment of asthma severity and the identification of distinct endotypes are essential for effective asthma management, particularly in patients with severe, treatment-refractory disease. These classifications are informed by both clinical presentation and biological markers, including ncRNA expression patterns. For example, the let-7 family and miR-98 expression levels were found to increase stepwise from mild to severe asthma, reflecting asthma severity. The lowest expression of these miRNAs was detected in patients with mild asthma, higher expression was observed in those with moderate asthma, and the highest expression was recorded in patients with severe asthma [115]. Regarding miRNAs specifically identifying eosinophils, regardless of asthma severity, two miRNAs were identified: miR-155 and miR-21. The expression of these miRNAs varied depending on the eosinophil count: the highest expression was observed in patients with eosinophilic asthma, and the lowest—in patients with noneosinophilic asthma [115]. Increased miR-155 expression has also been observed in the plasma of severe asthmatics compared to non-asthmatics or those with mild-to-moderate asthma [117]. Additionally, miR-185-5p has demonstrated the diagnostic power, not only distinguishing asthmatic from non-asthmatic individuals, but also scaling with disease severity and being most pronounced in patients admitted to intensive care units or those with adult-onset asthma, where clinical settings are frequently characterized by severe, steroid-refractory phenotypes [108].

### 4.2. Non-Coding RNAs and Corticosteroid Responsiveness in Asthma

Corticosteroids remain the mainstay of asthma treatment, but a subset of patients (estimated at 5–10%) exhibit corticosteroid resistance, which complicates disease management and increases the risk of severe outcomes [147]. Growing evidence suggests that alterations in ncRNA expression can directly affect the molecular pathways involved in airway inflammation, remodeling, and glucocorticoid sensitivity. For instance, it has been found that in severe asthma treated with corticosteroids, miR-146 expression decreases in CD8^+^ and CD4^+^ T cells [148].

One notable example of the lncRNA is PVT1 (plasmacytoma variant translocation 1), which has previously been implicated in the development and progression of diabetic nephropathy as well as breast, ovarian, and lung cancer [149,150,151]. Recent findings have extended the significance of PVT1 to airway inflammation: Austin et al. identified PVT1 as differentially expressed in airway smooth muscle cells from patients with non-severe and severe asthma, with higher PVT1 expression correlating with asthma severity and corticosteroid resistance [128]. It was also shown that targeting PVT1 modulates both cellular proliferation and IL-6 release (via transcription factor c-MYC regulation), highlighting its role in airway remodeling and steroid resistance. These data indicate that lncRNAs such as PVT1 may serve not only as disease biomarkers but also as functional regulators and potential therapeutic targets in asthma, particularly in severe phenotypes where treatment options remain limited.

Another key lncRNA is CASC7. Liu et al. reported that in airway smooth muscle cells from patients with severe asthma, the expression of the CASC7 was reduced. CASC7 contains a binding site for miRNA-21, which is implicated in PTEN/AKT signaling pathway; PTEN acts as a negative regulator of PI3K-dependent AKT signaling. In these airway smooth muscle cells, both miRNA-21 expression and AKT activity were elevated. Experimental modulation of CASC7 and miRNA-21, together with luciferase reporter assays, confirmed direct interactions among CASC7, miRNA-21, and PTEN mRNA. The data suggests that CASC7 enhances PTEN expression by repressing miRNA-21. Furthermore, overexpression of CASC7 inhibits the PI3K/AKT pathway, resulting in improved corticosteroid responsiveness [129]. However, further in vivo studies are needed to assess the combined effects of corticosteroids and CASC7 RNA for therapeutic purposes.

Furthermore, it was shown that, in the treatment of steroid-resistant asthma, lncRNA GAS5 acts as a glucocorticoid receptor, and pro-inflammatory mediators increase the amount of GAS5 both in airway smooth muscle cells and in the airway epithelium [130]. It was also demonstrated that reducing GAS5 expression decreased airway hyperresponsiveness. The same study showed that GAS5 regulates airway smooth muscle cell proliferation via miR-10a [131].

### 4.3. Distinct Non-Coding RNAs Signatures in Eosinophilic Versus Neutrophilic Asthma

Asthma can be classified into eosinophilic and neutrophilic endotypes based on the predominant inflammatory cells in the airways. Eosinophilic asthma is typically defined by >3% eosinophils in sputum, while neutrophilic is defined by >61% neutrophils. These endotypes are driven by distinct immunological pathways: eosinophilic asthma is associated with Th2 responses and type 2 cytokines (IL-4, IL-5, IL-13), whereas neutrophilic asthma involves Th1- and Th17-mediated inflammation [152,153]. Given these mechanistic differences, recent research has focused on identifying distinct ncRNA signatures associated with each endotype. The regulation of Th2 cells by ncRNAs is discussed in detail in the following subsection.

Regarding neutrophilic asthma, one illustrative example of ncRNA-mediated regulation is the function of lncRNA MEG3. MEG3 acts as a competing endogenous RNA (ceRNA) for miR-17, indirectly influencing the expression of transcription factors FoxP3 (essential for differentiation of regulatory T (Treg) cells) and RORγt (important for Th17 cell differentiation). This interaction disrupts the balance between Treg and Th17 cells [132]. Th17 cells promote inflammation by producing IL-17 and recruiting neutrophils, whereas CD4^+^CD25^+^ regulatory Treg cells suppress immune responses and help maintain immune homeostasis. An imbalance between these cell populations contributes to persistent airway inflammation in asthma [154].

In a transcriptome-wide analysis of whole blood from asthma patients with different endotypes, LNC_000127 was identified as a key modulator of Th2 inflammation, acting through the TCR/STAT/GATA3 signaling pathway. LNC_000127 expression was significantly upregulated in eosinophilic asthma compared to neutrophilic asthma and healthy controls. This suggests that LNC_000127 may enhance Th2 cytokine production and promote eosinophilic airway inflammation [133].

### 4.4. Non-Coding RNA Regulation of Th2 Polarization and Type 2 Immune Responses

ncRNAs are critical regulators of immune cell development, differentiation, and effector function. By modulating epigenetic, transcriptional, and post-transcriptional mechanisms, these molecules play essential roles in orchestrating type 2 immune responses, particularly Th2 polarization. Their regulatory networks are increasingly recognized as potential therapeutic targets in asthma and other allergic diseases.

One of the earliest studies identified miR-19a in CD4^+^ T cells as a stimulator of interleukin-13 (IL-13) secretion, a key cytokine in type 2 inflammation [118]. Previous studies of the miR-17-92 cluster, which includes miR-19a, showed that it promotes CD4^+^ T cell survival and proliferation [119]. The miR-17-92 cluster has also been shown to exert important functional effects on type 2 innate lymphoid cells (ILC2s). Deficiency of this miRNA cluster led to reduced cytokine production by ILC2s, diminished airway hyperresponsiveness, decreased mucus hypersecretion, and lower eosinophilic infiltration into the airways. Notably, in both ILC2s and Th2 cells, IL-13 production is regulated by miR-19a via shared targets, suggesting miR-19a as a promising therapeutic target for treating type 2 airway inflammation [120]. In addition, elevated miR-126 expression has been observed in patients with asthma. This miRNA indirectly increases the expression of the transcription factor GATA-3 in T cells, promoting the secretion of IL-5 and IL-13 by Th2 cells, which in turn activate eosinophils [121]. Conversely, several miRNAs have been reported to suppress Th2 cell activity. For instance, miR-24 and miR-27 inhibit IL-4 production in CD4^+^ T cells, and deletion of these miRNAs enhances Th2-dependent responses in vivo [122].

Notably, miR-371, miR-138, miR-544, miR-145, and miR-214 have been shown to regulate the Th1/Th2 balance by inhibiting Runx3, a transcription factor that promotes Th1 differentiation. In CD4^+^ T cells from asthmatic patients, Runx3 expression is reduced, contributing to the observed Th1/Th2 imbalance. Importantly, only the simultaneous inhibition of all five miRNAs was sufficient to restore Runx3 levels and correct the Th1/Th2 imbalance, underscoring the cooperative nature of ncRNA regulation in asthma [123].

In addition to miRNAs, lncRNA fantom3_9230106C11, highlighted through lncRNA–mRNA coexpression network analysis, showed high connectivity and regulatory potential. Its expression was significantly downregulated in both ex vivo and in vitro Th2 cells, implying a role in restraining Th2 differentiation. Computational predictions suggested that this lncRNA may interact with GATA1, a key transcription factor in Th2 lineage commitment, and miR-19, known for promoting IL-13 production. These interactions point toward a regulatory mechanism in which lncRNA fantom3_9230106C11 acts as a molecular scaffold or sponge, modulating the transcriptional and post-transcriptional control of Th2 immune responses and contributing to type 2 inflammation in asthma [134].

Other lncRNAs identified as regulators of Th2-type inflammation include MM9LINCRNAEXON12105+ and AK089315, which are coexpressed with genes involved in IL-4, IL-5, and IL-13 signaling, as well as Th2-associated transcription factors (STAT5, STAT6) and chemokines (CCL17, CCL22) in a murine model of asthma [135]. Additionally, lncRNA RP11-401.2 was upregulated in Th2 cells, which are closely associated with eosinophilic asthma [136]. Additional studies have shown that ENST00000444682, ENST00000566098, and ENST00000583179 are upregulated, while ENST00000579468 is downregulated in CD4^+^ T cells from asthmatic patients compared to healthy controls. These lncRNAs were significantly correlated with Th2-associated cytokines, such as IL-13, IL-5, IL-4, and IL-6, as well as with lung function (FEV_1_/FVC). Coexpression network analysis showed that these lncRNAs may modulate Th2 cell differentiation and pro-inflammatory cytokine production by interacting with genes involved in immune signaling pathways, including *SMAD7, WNT2B, C/EBP, T-bet*, and *NF-κB* [137]. Collectively, these findings underscore the multifaceted roles of ncRNAs in the regulation of Th2 polarization and type 2 immune responses in asthma, offering novel insights for therapeutic targeting.

### 4.5. Non-Coding RNAs in Airway Structural Cells and Remodeling

Airway remodeling, characterized by changes such as smooth muscle hypertrophy, hyperplasia, and epithelial–mesenchymal transition, is a hallmark of asthma and contributes to chronic airway obstruction and hyperresponsiveness. ncRNAs play essential roles in regulating the behavior of airway structural cells. For example, reduced miR-1 expression has been associated with bronchial smooth muscle cell hypertrophy in asthma [124]. miR-26a promotes hypertrophy of airway smooth muscle cells by targeting and downregulating the anti-hypertrophic kinase GSK-3β [125]. miR-221 regulates airway smooth muscle cell proliferation and IL-6 production [116], downregulation of the miR-200 family permits ZEB1/2 upregulation, driving epithelial–mesenchymal transition during airway remodeling in asthma [126]. In bronchial epithelial cells from asthmatic patients, elevated miR-155 expression has been reported [117]. Additionally, miR-146b-5p is highly expressed in airway smooth muscle cells from asthmatics upon stimulation [127].

lncRNAs have also emerged as critical regulators of airway remodeling. Functional studies using gain- and loss-of-function approaches have demonstrated that LNC_00882 expression is upregulated in airway smooth muscle cells following stimulation with platelet-derived growth factor, which in turn promotes cell proliferation. Mechanistically, luciferase reporter and RNA pull-down assays identified a direct interaction between LNC_00882 and miRNA-3619-5p, an miRNA that targets β-catenin. LNC_00882 has been shown to promote airway smooth muscle cell proliferation by enhancing Wnt/β-catenin signaling through sponging of miRNA-3619-5p, thus supporting its role in airway remodeling [138].

MALAT1, another lncRNA, has been elevated in airway smooth muscle cells after platelet-derived growth factor exposure and in neonatal asthmatic rat models [139,140]. Functional experiments have shown that silencing MALAT1 using siRNA in human airway smooth muscle cells, results in reduced cell proliferation and migration [140]. Mechanistic studies, including luciferase reporter assays, demonstrated that MALAT1 directly binds to miRNA-150. Since miRNA-150 targets the translation initiation factor 4E (eIF4E)—a key component of the AKT signaling pathway—MALAT1 indirectly activates AKT signaling by sponging miRNA-150, thereby promoting airway smooth muscle cell proliferation and migration, which contributes to airway remodeling in asthma [50]. Furthermore, MALAT1 knockdown in primary bronchial and tracheal smooth muscle cells was associated with decreased apoptosis and lower production of pro-inflammatory cytokines, involving the miRNA-133a–ryanodine receptor-2 (RyR2) regulatory axis [139].

Recent studies have also identified lncRNA NORAD as a regulator of epithelial and airway remodeling. NORAD was found to be significantly upregulated in TGF-β1-induced BEAS-2B human bronchial epithelial cells and in ovalbumin-challenged asthmatic mice. Experimental results revealed that NORAD acts as a sponge for miR-410-3p, regulating RCC2 and the Wnt/β-catenin signaling pathway, which in turn modulates epithelial–mesenchymal transition, airway remodeling, and inflammatory processes. Importantly, silencing NORAD was shown to attenuate airway remodeling and inflammation in vivo by reducing inflammatory cell infiltration and collagen deposition, suppressing IL-4, IL-13, TGF-β1, and immunoglobulin E production, as well as β-catenin and c-MYC expression, while increasing miR-410-3p expression [141]. These findings highlight the central role of ncRNAs in orchestrating airway structural changes and remodeling in asthma, operating through intricate and interconnected regulatory networks.

### 4.6. Regulation of Macrophage Polarization by Non-Coding RNAs in Type 2 Airway Inflammation

Recent findings highlight the critical involvement of lncRNAs in regulating macrophage polarization, particularly in promoting or suppressing M2-type responses associated with type 2 inflammation in asthma. LncRNA AK085865 was shown to drive M2 macrophage polarization in a murine asthma model induced by *Dermatophagoides farinae* allergen. Knockout of AK085865 alleviated airway inflammation, reduced IgE levels, and decreased both M2 macrophages and eosinophil infiltration. Mechanistically, AK085865 promotes M2 polarization, which in turn enhances ILC2 differentiation via exosomal signaling, thereby amplifying type 2 inflammation [142]. Similarly, lncRNA BAZ2B was found to be upregulated in asthmatic children and promoted M2 macrophage activation by stabilizing BAZ2B pre-mRNA, increasing BAZ2B expression, and downstream interferon regulatory factor 4 (IRF4) activity. Knockdown of either lnc-BAZ2B or BAZ2B reduced inflammation in a cockroach allergen-induced asthma model, suggesting therapeutic potential [143]. In contrast, lncRNA PTPRE-AS1 acts as a negative regulator of M2 polarization. It enhances PTPRE expression by promoting H3K4 trimethylation of its promoter via interaction with WDR5. Loss of PTPRE-AS1 in asthmatic mice led to exaggerated M2 macrophage responses and more severe airway inflammation. Importantly, both PTPRE-AS1 and PTPRE levels were reduced in peripheral blood mononuclear cells from asthmatic patients, supporting their potential as biomarkers and negative regulators of M2-mediated inflammation [144].

### 4.7. PIWI-Interacting RNAs as Novel Modulators in Asthma Pathogenesis

Although piRNAs are traditionally known for their role in germline genome integrity, recent findings suggest their involvement in immune regulation and asthma pathogenesis [145]. It was found that piR-37213, piR-31038, piR-33520, and piR-34021 were associated with both eosinophil counts and IgE levels, suggesting a role in allergic inflammation. piR-43770 was also associated with total serum IgE. Several piRNAs (piR-33487, piR-36063, piR-32571) were also associated with eosinophils count at baseline, whereas piR-58469, piR-43768, and piR-43770—with serum IgE level. In the study, certain piRNAs were mapped to genes or genomic regions functionally linked to asthma. piR-33064, located in an intron of the *PARD3B* gene, may influence asthma pathogenesis by affecting epithelial tight junction integrity, a key element in airway barrier dysfunction [145,146]. piR-57460 lies within BCL2L1-AS1, an antisense lncRNA of the *BCL2L1* gene, which regulates mitochondrial apoptosis—a process implicated in eosinophilic inflammation and airway remodeling [145,155]. Moreover, piR-58469, piR-36707, and piR-35549, found in mitochondrial genome regions, were linked to serum IgE and point toward mitochondrial dysfunction as a contributing mechanism in asthma. Furthermore, enrichment analyses revealed that host genes of eosinophil- and IgE-associated piRNAs are involved in thymus development and T cell proliferation, indicating a broader role for piRNAs in adaptive immunity [145]. These data collectively suggest that piRNAs may act through various molecular pathways to influence T2-high asthma phenotypes. As such, they represent a novel and promising class of ncRNAs for the discovery of asthma biomarkers and mechanistic research.

### 4.8. Metabolite-Mediated Effects of miRNAs in Asthma Pathogenesis

Recent integrative omics studies have revealed that specific asthma-associated miRNAs may influence disease phenotypes through interactions with metabolic intermediates that participate in key immunological and inflammatory pathways. Taurine, a semi-essential amino acid and potent antioxidant, inhibits pro-inflammatory cytokines (IL-1, IL-6, and TGF-β) and is elevated in plasma and bronchoalveolar lavage fluid of asthma patients [156,157]. Several miRNAs, including miR-96-5p, miR-1271-5p, miR-27a-3p, miR-21-3p, and miR-125b-2-3p, have been identified as regulators of *GAD1* and *GAD2* genes, which encode the glutamate decarboxylase enzyme to catalyze taurine biosynthesis, indicating a potential mechanism by which ncRNAs indirectly affect inflammation via taurine regulation [158,159].

Similarly, 12,13-diHOME (a metabolite derived from linoleic acid via cytochrome P450 (CYP2J and CYP2C), and soluble epoxide hydrolase (sEH)) has been linked to immune dysregulation and an increased risk of asthma [160]. Elevated levels of 12,13-diHOME have been observed in individuals with asthma. This metabolite has been shown to mediate the effects of at least 19 asthma-relevant miRNAs. Notably, seven of these miRNAs (hsa-miR-125a-5p, hsa-miR-29a-3p, hsa-miR-122-5p, hsa-miR-345-5p, hsa-miR-21-3p, hsa-miR-93-3p, and hsa-miR-146b-3p) target genes involved in the linoleic acid metabolism pathway, thereby potentially influencing the synthesis of 12,13-diHOME itself and modulating downstream pro-inflammatory lipid mediators [158].

Another metabolite of interest is 9-cis-retinoic acid, a vitamin A derivative that binds to and activates nuclear retinoid acid and retinoid X receptors, exerting anti-inflammatory effects [161,162]. Levels of this metabolite differ between asthmatics and healthy controls [163]. It has revealed that hsa-miR-16-5p, hsa-miR-30d-5p, hsa-miR-125b-5p, and hsa-miR-27b-3p regulate key enzymes (ALDH1A1, ALDH1A3) in the retinoic acid biosynthetic pathway, further suggesting miRNA involvement in airway inflammation regulation through this vitamin A metabolite [158].

## 5. Prospects of ncRNA-Based Therapies in Asthma

In 2018, the U.S. Food and Drug Administration (FDA) approved the first siRNA-based drug, patisiran (brand name Onpattro), for the treatment of hereditary transthyretin-mediated amyloidosis (hATTR) in adults. Patisiran works by silencing the gene encoding the misfolded transthyretin protein, the underlying cause of hATT. This event set a precedent and paved the way for RNA-based therapeutics [164]. However, according to the most recent data, there are currently no approved ncRNA-based drugs or ongoing clinical trials specifically targeting asthma, although several preclinical studies in animal models are underway.

For example, a successful preclinical study targeted miR-21. In a murine model of allergic asthma, intranasal administration of an antagomir against miR-21 selectively downregulated miR-21 expression in lung tissues. This intervention led to a significant reduction in airway hyperresponsiveness, decreased total and eosinophil cell counts in bronchoalveolar lavage fluid, and lower levels of Th2 cytokines (IL-4, IL-5, IL-13). Mechanistically, the miR-21 antagomir shifted the Th1/Th2 balance by increasing IL-12 expression and modulating key transcription factors involved in T cell polarization, including STAT, GATA, and T-bet. Collectively, these findings demonstrate that miR-21 antagomir therapy can effectively suppress allergic airway inflammation by inhibiting Th2 activation, supporting its potential as a therapeutic strategy for asthma [165].

By contrast, a less successful example was reported by Plank et al., who demonstrated that intranasal administration of an antagomir targeting miR-155-5p in a murine asthma model specifically reduced miR-155-5p levels in lung tissues but did not affect disease phenotypes such as airway inflammation or hyperresponsiveness. The lack of therapeutic effect was attributed to inefficient antagomir delivery to CD4+ T cells, despite successful knockdown in macrophages and monocytes. This study underscores both the high specificity and current limitations of ncRNA-based interventions, highlighting the critical need for cell-specific delivery strategies in the development of effective ncRNA therapeutics for asthma [166].

Liao et al. applied ceRNA network construction and database screening to identify lncRNAs that may regulate asthma-related gene expression by sponging specific miRNAs. Their study identified five lncRNAs—MALAT1, MIR17HG, CASC2, MAGI2-AS3, and DAPK1-IT1—as key regulators within these networks. Further, they predicted eight candidate drugs, including tamoxifen, ruxolitinib, tretinoin, quercetin, dasatinib, levocarnitine, niflumic acid, and glyburide, that could potentially interact with these lncRNA-miRNA-mRNA axes [167]. In addition, a few preclinical studies have investigated specific compounds that modulate the lncRNA/miRNA/mRNA axis to attenuate airway remodeling and inflammation in asthma models. For instance, Schisandrin B, a natural compound derived from *Schisandrae*, has been shown to inhibit airway smooth muscle cell proliferation and migration in asthmatic rat models by downregulating the lncRNA BCYRN1 and upregulating miR-150. Mechanistically, Schisandrin B’s effects were reversed by an miR-150 inhibitor, indicating its action through the BCYRN1/miR-150 pathway [168]. Similarly, α-Asarone, an herbal compound from *Acorus gramineus*, suppressed airway smooth muscle cell proliferation and reduced levels of Th2 cytokines (IL-4, IL-5, IL-13) in respiratory syncytial virus-induced asthmatic rats, in part by downregulating the lncRNA PVT1. Since PVT1 acts as a ceRNA that sponges miR-203a and blocks its inhibition of E2F3 (E2F transcription factor 3, which promotes airway smooth muscle cell activation, proliferation, and migration), reduction in PVT1 allows miR-203a to suppress E2F3 expression, thereby attenuating airway smooth muscle cell activation and airway remodeling [169]. Additionally, the commonly used drug for treating cough-variant asthma, montelukast sodium, was recently shown to reduce the inflammatory factor IL-4 and increase the anti-inflammatory factor interferon (IFN)-γ, thereby enhancing pulmonary function. In ovalbumin-induced asthmatic mice, lncRNA PCGEM1 was downregulated, whereas overexpression of PCGEM1 further enhanced the anti-inflammatory effects of montelukast, likely through inhibition of the nuclear factor-κB (NF-κB) pathway [170].

Despite promising preclinical studies demonstrating the therapeutic potential of ncRNA-targeting strategies in asthma, no ncRNA-based drugs have yet reached clinical trials for this disease. The main obstacles include the challenge of delivering these therapeutics specifically to airway cells, ensuring sufficient stability and specificity, and minimizing off-target effects and toxicity. Advances in delivery technologies, molecular modifications, and comprehensive preclinical validation are essential to translate these approaches into clinical use. Thus, while the field holds significant promise, further research is required to overcome these hurdles and realize the clinical potential of ncRNA-based therapies for asthma.

## 6. Limitations

Despite providing a comprehensive overview of current knowledge regarding the roles of ncRNAs in eosinophil biology and asthma pathogenesis, this review has several limitations. Firstly, most of the evidence summarized here is derived from preclinical studies, including in vitro experiments and animal models, and therefore may not fully reflect human disease mechanisms. Secondly, while several functional mechanisms by which specific ncRNAs regulate asthma pathogenesis are discussed, many molecular pathways remain incompletely understood due to the novelty and technical complexity of ncRNA research—including challenges related to low expression levels, cell-type specificity, and the multi-layered regulation of ncRNAs. Additionally, a lack of standardization in ncRNA research, such as differences in experimental design, sample processing, and data analysis, complicates cross-study comparisons and limits reproducibility. Furthermore, many ncRNAs still lack clear functional annotation or experimentally validated targets. Importantly, although the clinical potential of ncRNAs as biomarkers or therapeutic targets for asthma is promising, it remains unproven in large-scale, longitudinal human studies. Finally, this is a rapidly evolving research field, and new findings could substantially alter current understanding, highlighting the need for ongoing review and continuous integration of emerging data.

## 7. Conclusions

Although only a small fraction of the human genome encodes proteins, approximately 76% is transcribed into RNA, and most of these transcripts are ncRNAs. Once dismissed as transcriptional noise, ncRNAs have emerged as central regulators of cellular function, orchestrating genome organization, stability, and gene expression. In the context of asthma, a heterogeneous and increasingly prevalent disease, ncRNAs represent a crucial yet underexplored layer of molecular regulation.

Many ncRNAs display disease-specific expression patterns and modulate both immune and structural cell behavior through diverse mechanisms. These properties underscore the value of ncRNAs not only as mechanistic drivers of disease but also as promising, accessible biomarkers for asthma phenotyping, early disease detection, and monitoring of disease progression, as well as potential therapeutic targets, particularly given their stability in exosomes and extracellular fluids. Although no ncRNA-based drugs have yet reached clinical application in asthma, emerging evidence from cellular and animal studies highlights their prognostic value and potential for guiding personalized treatment strategies. ncRNAs involvement in corticosteroid responsiveness, airway remodeling, and immune signaling in airway inflammation highlights their relevance for future research and the development of personalized treatment strategies. However, their participation in complex and overlapping regulatory networks presents both opportunities and significant challenges for clinical translation. Therefore, large-scale, longitudinal studies encompassing diverse asthma endotypes are essential to fully realize the potential of ncRNAs as next-generation biomarkers and therapeutic targets in personalized asthma care.

## Data Availability

No new data were created or analyzed in this study. Data sharing is not applicable to this article.
